# Effects of the Proactive interdisciplinary self-management (PRISMA) program on self-reported and clinical outcomes in type 2 diabetes: a pragmatic randomized controlled trial

**DOI:** 10.1186/s12902-019-0466-0

**Published:** 2019-12-11

**Authors:** Esther du Pon, Nanne Kleefstra, Frits Cleveringa, Ad van Dooren, Eibert R. Heerdink, Sandra van Dulmen

**Affiliations:** 1 Research Group Process Innovations in Pharmaceutical Care, Utrecht University of Applied Sciences, PO Box 12011, 3501 AA Utrecht, the Netherlands; 2 Diabetes Centre, Isala, Zwolle, the Netherlands; 3 Medical Research Group, Langerhans, Ommen, 7731 MX the Netherlands; 4 Department of GGZ Drenthe research and High Intensive Care, GGZ Drenthe mental health services, Assen, 9404 LA the Netherlands; 5 Department of Internal Medicine, University of Groningen and University Medical Center Groningen, Groningen, 9713 GZ the Netherlands; 6 Julius Center for Health Sciences and Primary Care, University Medical Center Utrecht, Utrecht, 3584 CX the Netherlands; 7 Utrecht Institute for Pharmaceutical Sciences, Utrecht University, Utrecht, the Netherlands; 8 Nivel (Netherlands institute for health services research), Utrecht, 3513 CR the Netherlands; 9 Department of Primary and Community Care, Radboud university medical center, Radboud Institute for Health Sciences, Nijmegen, 6525 GA the Netherlands; 10 Faculty of Health and Social Sciences, University of South-Eastern Norway, 3045 Drammen, Norway

**Keywords:** E-health, Type 2 diabetes, Primary care, Group education, Self-management

## Abstract

**Background:**

Diabetes self-management education can be helpful for patients with type 2 diabetes in managing their condition. We aimed to study the effects of the group-based PRoactive Interdisciplinary Self-MAnagement (PRISMA) training program on self-reported and clinical outcomes in patients with type 2 diabetes treated in general practice.

**Methods:**

Persons aged 18 years or older diagnosed with type 2 diabetes and treated in primary care were included. In a randomized controlled trial design (1:1), patients were followed for 6 months with an extension phase of 6 months. Block randomization was used. The patients with type 2 diabetes received either PRISMA in addition to usual care or usual care only. All patients completed a range of validated questionnaires (including knowledge, skills, and confidence for self-management [PAM], diabetes self-care behavior [SDSCA], health-related quality of life [EQ-5D], and emotional well-being [WHO-5]). In addition, clinical outcomes (HbA1c, body mass index, systolic blood pressure, and cholesterol levels) were collected during the routine diabetes checkups.

**Results:**

Of the total sample (*n* = 193), 60.1% were men. The mean age was 69.9 years (SD = 9.1). No significant differences were found on self-reported outcomes between the groups at 0, 6, and 12 months. The clinical outcomes were not reported due to a large number of missing values.

**Conclusion:**

PRISMA did not improve self-reported outcomes in patients with type 2 diabetes treated in primary care. It was not possible to make a statement about the clinical effects.

**Trial registration:**

date: 16/07/2014, number: NL4550 (https://www.trialregister.nl/trial/4550).

## Background

In the Netherlands, 66 in 1000 persons were diagnosed with type 2 diabetes mellitus in 2017 [1]. This rate is expected to increase to 80 in 1000 persons by 2025 [2]. There will be no comparable increase of health care providers, which compromises the availability of face-to-face time between patients and health care providers. In the Dutch health system, 95% of patients with type 2 diabetes are treated in primary care by a general practitioner (GP) and a practice nurse (PN) [3]. To lower the risk of cardiovascular complications and manage the increasing number of type 2 diabetes patients, encouraging self-management could be part of the solution. Self-management can be defined as the active participation of patients in their treatment [4] to minimize the impact of type 2 diabetes on their physical health and functioning and help them to cope with the psychological effects of their illness [5]. In the Netherlands, the quality of care for patients with type 2 diabetes has improved considerably in recent decades [6]. In 1998, 55% of patients with type 2 diabetes younger than 75 years had an HbA1c higher than 53 mmol/mol. In 2013, this decreased to 29% [5]. For patients of 75 years and older, similar trends were found for HbA1c. Poor health behavior places patients with type 2 diabetes at an increased risk of disease progression, impacting their quality of life and increasing their risk of additional health problems and premature death [7–9].

The effectiveness of diabetes self-management ultimately depends on patients’ adherence with lifestyle recommendations and treatment [10]. Patients need to understand the principles and importance of self-management activities [11], which makes diabetes self-management education a key component of diabetes care. Diabetes self-management education can be helpful for patients with type 2 diabetes in managing their condition [12]. It aims not only to enhance the patients’ medical understanding but also to improve their intrinsic motivation, belief in their innate ability to achieve goals (self-efficacy), illness perception, self-management skills, and behavior [13]. Moreover, a recent systematic review showed that diabetes self-management education resulted in improved HbA1c [14]. Group-based diabetes self-management education seems to be more effective than usual care and individual education at improving clinical, lifestyle, and psychosocial outcomes [15].

One example of an evidence-based diabetes self-management education program is the group-based Proactive Interdisciplinary Self-MAnagement (PRISMA) training program. This program is based on the DESMOND (Diabetes Education and Self-Management for Ongoing and Newly Diagnosed) program, which was developed and tested in the UK [12, 16, 17]. DESMOND has proven to be (cost-)effective in patients newly diagnosed with type 2 diabetes [16, 17]. PRISMA was adapted for use in type 2 diabetes in primary care in the Netherlands and, in a previous study, seemed to improve self-management behavior in terms of dietary behaviors, foot care, and action planning three months posttraining [18]. However, the effects of PRISMA on self-reported and clinical outcomes in a controlled setting are still unknown. Therefore, the present study aimed to investigate the effects of PRISMA on self-reported (knowledge, skills and confidence for self-management, diabetes self-care behavior, health-related quality of life, and emotional well-being) and clinical outcomes in patients with type 2 diabetes treated in general practice.

## Methods

### Study design

The current study is part of the Diabetes Education and Self-Management to Increase Empowerment (DESTINE) study, described in detail elsewhere [19]. DESTINE has a randomized controlled trial (RCT) design (1:1) in which patients are followed for 6 months with an extension phase of another 6 months. The study has a pragmatic character, as it was carried out in daily practice. More details about the DESTINE study can be found in a previous article [20]. Persons of 18 years and older diagnosed with type 2 diabetes and treated in primary care were included. Eight general practices in the eastern part of the Netherlands participated, and eligible patients were informed and asked to participate by GPs. Block randomization was used to allocate participants to one of the two groups. The participants were randomized in 10 blocks of 20 participants each. All criteria of the CONSORT checklist were reported (see Checklist S1) [21].

### Description of the intervention

Patients with type 2 diabetes received either PRISMA in addition to usual care or usual care only. According to the guidelines of the Dutch College of GPs (NHG-Standaard), usual care involves two to four visits per year with the PN and at least one annual check-up with the GP. In addition, all patients had access to an online care platform (e-Vita), which aimed to support patients’ self-management skills. The PRISMA program consisted of two group meetings about type 2 diabetes, guided by different PNs and a dietician specialized in diabetes care. These trainers strictly adhered to the PRISMA protocol. In short, the following topics were discussed: blood glucose levels, medication, nutrition, physical activity, complications, and personal risk factors. The PRISMA program has been described in detail previously [20]. The PRISMA philosophy is to actively involve patients and support them with the regulation of the disease. PRISMA aims to support patients by making them aware of their specific risk profile regarding the development of complications and providing information about methods to decrease their risks. Through a constructive approach, a learning process is initiated that helps patients to (continue to) work on promoting and monitoring their health. The trainers encouraged the patients to discuss their individual action plans with their health care provider during their next consultation and bring up the topics important to them. The two PRISMA meetings should therefore be seen as a starting point to motivate/activate patients, with behavior change as the final objective.

### Outcomes

The outcomes of the study included self-reported data (questionnaires), as well as clinical data retrieved from the GP information systems.

#### Self-reported data

All patients completed a range of validated questionnaires at T0 (0 months, at the end of two PRISMA meetings), T1 (6 months) and T2 (12 months). The following questionnaires were used:

1. The Patient Activation Measure (PAM). The PAM is a validated 13-item instrument used to assess the knowledge, skills, and confidence for self-management [22]. Each item has four response categories with scores from 1 (strongly disagree) to 4 (strongly agree). Higher scores represent higher levels of patient activation.
2. The Summary of Diabetes Self-Care Activities (SDSCA) scale. This scale assesses diabetes self-care behavior over the previous 7 days in six domains: diet, exercise, self-monitoring of blood glucose, foot care, adherence and smoking [23]. The mean score of each item was reported [24].
3. The EuroQol Five Dimension (EQ-5D-3 L) scale. This questionnaire assesses health-related quality of life and consists of two parts: the EQ-5D descriptive system and the EQ visual analog scale (EQ-VAS) [25]. The EQ-5D-3 L comprises five dimensions: mobility, self-care, usual activities, pain/discomfort, and anxiety/depression. Each dimension has three levels: no problems, some problems, and extreme problems. The EQ-5D-3 L includes a visual analog scale (EQ-VAS) where individuals rate their own health today on a scale from 0 (worst imaginable health) to 100 (best imaginable health).
4. The World Health Organization Well-being Index 5-Item (WHO-5) scale. The WHO-5 assesses emotional well-being and covers five items: subjective quality of life based on positive mood (good spirits, relaxation), vitality (being active and waking up fresh and rested), and general interest (being interested in things) [26]. Each of the five items is rated on a 6-point Likert scale from 0 (not present) to 5 (constantly present). Higher scores represent higher levels of emotional well-being.

Additional demographics (gender, age, education level, and type 2 diabetes duration) were included as patient characteristics.

#### Clinical outcomes

The clinical outcomes described in the national primary care guideline for diabetes treatment were included in the study: HbA1c, body mass index (BMI), systolic blood pressure, and cholesterol levels (total cholesterol, high-density lipoprotein [HDL], and low-density lipoprotein [LD]). These clinical outcomes were collected during the routine diabetes checkups as part of usual care. These data were individually linked to our own database and anonymized. The data were collected at T0 (0 months), T1 (6 months), and T2 (12 months). For T0, only data available no more than 4 months before the intervention were used. For T1 and T2, only data 6 weeks before and 6 weeks after the measuring moment were used. Using a research identification number for every patient, all data were collected and analyzed anonymously.

### Sample size calculation

A power calculation was carried out on the primary outcome measure (usage of the e-Vita online care platform), resulting in 81 participants needed per group [19]. The usage of e-Vita was investigated by du Pon et al. (2019 – submitted) in another study.

### Statistical analysis

All analyses were conducted using IBM SPSS Statistics version 22. Normally distributed data are presented as the means and standard deviation, while skewed data are presented as the median and interquartile range. Dichotomous/categorical data are presented as the numbers and percentage of the total. To evaluate differences in target variables between groups, a *t*-test was used if the data were normally distributed; if not, a Mann-Whitney U test was used. An intention to treat analysis was conducted.

## Results

**Fig. 1:**
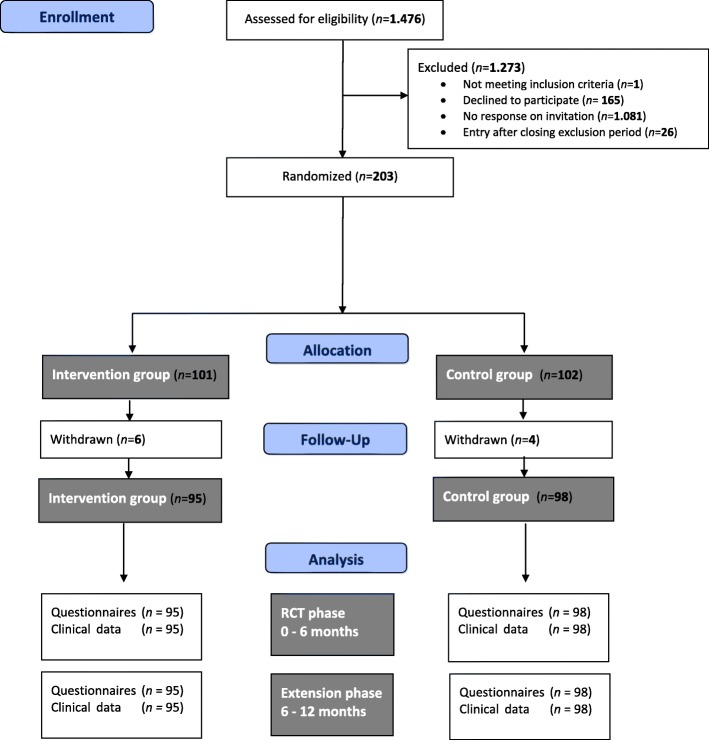
Flow chart of the randomized controlled trial. RCT = randomized controlled trial

The inclusion period lasted 9 months (June 2014 to February 2015). Of 1476 invited patients with type 2 diabetes treated at participating general practices, 203 (13.8%) were included in the study and signed the informed consent form: 101 patients were randomized in the intervention group; and 102, in the control group. After inclusion, 10 patients (4.9%) withdrew from the study: 6 in the intervention group and 4 in the control group. Patients withdrew because of illness, immigration, or personal reasons. Therefore, 95 patients in the intervention group and 98 patients in the control group were analyzed. In the intervention group, 68 (71.6%) of 95 patients attended at least one of the two PRISMA meetings in the RCT phase. The patient flow chart is presented in Fig. 1 [21].

### Patient characteristics

The patient characteristics are presented in Table 1. Of the total sample (*n* = 193), 60.1% were men. The mean age was 69.9 ± 9.1 years (range, 35–96).

**Table 1:** Baseline patient characteristics (*n* = 193)

*n (%)/mean ± SD/median (25–75 quartiles)*	Intervention group (*n* = 95)	Control group (*n* = 98)
Male	56 (58.9)	60 (61.2)
Age, years	69.7 ± 9.8	70.1 ± 10.1
Education level^a^		
Low Moderate High Unknown	4 (4.2) 41 (43.2) 12 (12.6) 38 (40.0)	8 (8.2) 45 (45.9) 11 (11.2) 34 (34.7)
Type 2 diabetes duration, years	6 (4–8)	6 (4–9)

^a^Low = no education or primary education; moderate = lower secondary education, (upper) secondary education or post-secondary non-tertiary education (including vocational education); high = tertiary education (bachelor’s degree or higher)

#### Self-reported outcomes

Table 2 shows the scores on knowledge, skills, and confidence in self-management (PAM), diabetes self-care behavior (SDSCA), health-related quality of life (EQ-5D), and emotional well-being (WHO-5) of the intervention and control groups at 0, 6, and 12 months. No significant differences were found between the groups.

**Table 2:** Scores on clinical outcomes of the intervention and control groups at 6 and 12 months

*n* (%)/mean ± SD/median (25–75 quartiles)	Baseline–0 months				RCT phase–6 months						Extension phase–12 months					
	Intervention group (*n* = 95)		Control group (*n* = 98)		Intervention group (*n* = 95)		Control group (*n* = 98)				Intervention group (*n* = 95)		Control group (*n* = 98)			
		Analyzed (*n*)		Analyzed (*n*)		Analyzed (*n*)		Analyzed (*n*)	Mean / median^a^ difference (95% CI)	*p*-value		Analyzed (*n*)		Analyzed (*n*)	Mean / median^a^ difference (95% CI)	*p*-value
PAM sum score higher scores represent higher levels of patient activation (range 13–52)	40.88 ± 5.48	57 (60)	41.95 ± 5.08	64 (65.3)	40.07 ± 5.76	59 (62.1)	41.78 ± 5.38	59 (60.2)	0.63 (−1.77–3.03)	0.60	40.53 ± 5,36	47 (49.5)	41.20 ± 5.27	44 (44.9)	0.019 (−2.23–2.28)	0.99
SDSCA diabetes self-care behavior in days a week (range 0–7)																
General diet	6.00 (4.75–7.00)	57 (60)	5.50 (4.75–6.75)	57 (58.2)	6.00 (5.00–7.00)	59 (62.1)	6.00 (5.00–7.00)	57 (58.2)	− 0.08^a^ (− 0.84–1.00)	0.61	6.00 (5.00–7.00)	46 (48.4)	6.00 (5.00–7.00)	43 (43.9)	0.03 ^a^ (− 0.63–0.74)	0.20
Specific diet	5.50 (4.50–6.00)	60 (63.2)	6.00 (5.00–6.50)	60 (61.2)	5.50 (4.60–6.00)	60 (63.2)	5.50 (4.50–6.30)	58 (59.2)	−0.06^a^ (− 0.63–0.74)	0.55	5.50 (4.50–6.00)	49 (51.6)	5.5 (5.00–6.50)	45 (45.9)	− 0.09^a^ (− 0.67–0.86)	0.92
Exercise	5.00 (3.00–6.00)	59 (62.1)	4.50 (2.90–6.00)	62 (63.3)	4.00 (3.00–5.40)	60 (63.2)	4.00 (3.00–6.00)	57 (58.2)	0.13^a^ (− 1.09–0.83)	0.17	4.50 (2.50–6.00)	49 (51.6)	5.00 (3.50–6.50)	43 (43.9)	− 0.21^a^ (− 1.28–1.20)	0.21
Blood glucose control	0.00 (0.00–0.50)	59 (62.1)	0.00 (0.00–0.50)	62 (63.3)	0.00 (0.00–0.50)	61 (64.2)	0.00 (0.00–0.50)	59 (60.2)	0.11^a^ (−2.70–0.97)	0.49	0.00 (0.00–0.88)	48 (50.5)	0.00 (0.00–0.50)	44 (44.9)	0.00^a^ (− 0.06–0.06)	0.30
Foot care	1.00 (0.00–3.50)	57 (60)	1.00 (0.00–3.30)	61 (62.2)	1.00 (0.00–3.50)	58 (61.1)	1.75 (0.00–3.50)	58 (59.2)	0.31^a^ (−2.58–0.46)	0.78	1.50 (0.00–4.13)	50 (52.6)	1.25 (0.00–3.50)	44 (44.9)	− 0.26^a^ (− 1.20–2.21)	0.04
Medication	7.00 (7.00–7.00)	58 (61.1)	7.00 (7.00–7.00)	59 (60.2)	7.00 (7.00–7.00)	56 (58.9)	7.00 (7.00–7.00)	55 (56.1)	−0.18 (− 0.53–0.16)	0.30	7.00 (7.00–7.00)	48 (50.5)	7.00 (7.00–7.00)	39 (39.8)	−0.51 (− 1.11–0.09)	0.07
Smoking number of patients who smoked in the last 7 days	7 (14.2)	49 (51.6)	4 (7.1)	60 (61.2)	9 (15.5)	59 (62.1)	6 (11.8)	51 (52.0)	0.02 (−0.06–0.10)	0.62	7 (14.9)	47 (49.5)	4 (8.9)	45 (45.9)	0.06 (−0.02–0.14)	0.16
EQ-5D index score higher scores represent higher levels of health-related quality of life (range 0–1)	0.89 (0.84–1.00)	58 (61.1)	0.89 (0.84–1.00)	62 (63.3)	0.84 (0.77–1.00)	63 (66.3)	0.84 (0.78–1.00)	61 (62.2)	− 0.00 (− 0.06–0.05)	0.92	0.89 (0.81–1.00)	48 (50.5)	0.88 (0.79–1.00)	46 (46.9)	0.01 (− 0.68–0.09)	0.76
EQ-VAS higher scores represent higher levels of health-related quality of life(range 0–100)	80.00 (71.30–90.00)	56 (58.9)	80.00 (70.00–90.00)	56 (57.1)	80.00 (65.00–90.00)	57 (60)	80.00 (70.00–86.30)	44 (55.1)	−8,11 (− 15.01– − 1.22)	0.02	80.00 (70.00–90.00)	43 (45.3)	80.00 (79.50–90.00)	42 (42.9)	−6.65 (− 15.27–-1.21)	0.13
WHO-5 index score higher scores represent higher levels of emotional well-being (range 0–100)	76.00 (61.00–80.00)	56 (58.9)	80.00 (72.00–80.00)	57 (58.2)	76.00 (57.00–80.00)	56 (58.9)	72.00 (64.00–80.00)	55 (56.1)	−3.90^a^ (−.07–15.87	0.93	78.00 (65.00–80.00)	42 (44.2)	76.00 (68.00–80.00)	39 (39.8)	−5.21 (− 13.88–3.47)	0.23

SD = standard deviation; RCT = randomized controlled trial; PAM = patient activation measure; SDSCA = summary of diabetes self-care activities; EQ-5D = euroqol five dimension; EQ-VAS = euroqol visual analog; WHO-5 = world health organization well-being index 5-item

### Clinical outcomes

The clinical outcomes (HbA1c, BMI, systolic blood pressure, and cholesterol [total, HDL, LDL]) were not reported in this article due to a large number of missing values. The missing values varied from 41.8 to 88.8%. Therefore, it is not possible to make a statement about the clinical effects. The clinical outcomes are available in an appendix.

## Discussion

We hypothesized that offering the PRISMA program would result in better self-reported and clinical outcomes in patients with type 2 diabetes treated in primary care. However, no effects were found on the following self-reported outcomes: knowledge, skills and confidence for self-management, diabetes self-care behavior, health-related quality of life, and emotional well-being. In addition, it was not possible to make a statement about the clinical effects.

Previous observational research showed that PRISMA appeared to improve self-management behavior in terms of dietary behaviors, foot care, and action planning after 3 months [18]. The lack of effects in self-reported outcomes in our study may be explained by the already high scores at baseline. These high scores indicated that the patients included in our study had generally well managed type 2 diabetes, so there was limited room for improvement. In addition, Van Vught et al. (2016) observed patients for only 3 months. Changes in outcomes could have been diminished after 6 or 12 months. However, PRISMA was expected to change self-management skills by achieving personal goals (e.g., losing weight), which usually would be visible in clinical measures in the intermediate term or longer. Therefore, in our study, 6 and 12 months were logical terms. Diabetes self-management education interventions appear most effective when group and individualized interventions are combined [27]. Therefore, patients probably need additional encouragement to change their behavior, such as with follow-up education in either group or individual settings.

Some evidence also suggests that contact exceeding 10 h for diabetes self-management education interventions are more likely to result in additional decreases in HbA1c [14]. However, PRISMA consisted of two interactive group meetings totaling 7 h [12]. Thus, additional meetings may have been needed. However, the day-to-day management of type 2 diabetes requires major responsibility from patients. Group education (combined with access to an online care platform) would be an extra activity and, as a result, patients could become overwhelmed. Moreover, our target group might not be interested in this type of interventions, which is the reality of usual care.

A strength of this study was that it was embedded in routine primary diabetes care, which means that it was designed to test PRISMA in the full spectrum of everyday usual care to maximize applicability and generalizability. Furthermore, randomization was performed at a patient level, which generally decreases influences of health care providers. Some limitations of this study must be mentioned as well. First, the eight participating general practices diverged in the inclusion rate: of the 1476 invited patients, most were registered in three general practices. Thus, the extent to which the health care providers motivated their patients to participate could have played a role. However, the characteristics of the patients (sex, age, education) do reflect the general Dutch population with type 2 diabetes. Second, in spite of the expected positive effects, in other studies about 30–50% of the eligible patients do not participate in diabetes education [28, 29]. In our study this rate was even lower. Despite our efforts to enthuse patients about the PRISMA program, only 12% of the approached patients participated. Therefore, selection bias was very likely. Patients who experience difficulties with self-management behavior probably do not voluntarily take part in studies or do not show up at interventions. Moreover, recruiting from a clinical sample may exclude the patients in greatest need of self-management, i.e. people who actually do not visit a general practice. Our target group may have been uninterested in this type of intervention, which could explain our low participation rate. If especially motivated patients participated, this could also explain why the self-reported and clinical measures were already quite good. However, despite their motivation, 28% of the patients from the intervention group who agreed to participate in the study did not attend at least one meeting of PRISMA. Patients changed their mind because were persuaded to enroll by their healthcare provider despite lack of interest. Patients were invited for PRISMA per letter signed by their own GP. Possibly, more personal attention in terms of reminding patients by telephone a couple of days before PRISMA would have prevented dropouts. Third, in an attempt to avoid a type III error, we monitored as many process factors as possible during the implementation of PRISMA. A type III error occurs when evaluating a program that has not been adequately implemented [30]. Such low intervention fidelity could decrease the interpretability of the data collected. As mentioned, PRISMA was guided by different PNs and dieticians, and their work experience in the diabetes field varied. However, all trainers used the same protocol and reported deviations from this protocol after the training. Notable deviations were not reported. In an ideal situation, the researcher would personally attend or record all training sessions in order to check intervention fidelity, however, this was not possibly due to a lack of time. Fourth, the great number of missing values in the clinical outcomes should be acknowledged. As a result, it is not possible to make a statement about the clinical effects. This could be explained by the fact that the study was carried out in daily practice. The clinical outcomes were collected annually during the routine diabetes checkups as part of usual care, and no extra laboratory tests were done. As a result, we were dependent on the data delivered as part of usual care. The missing values in the self-reported data were due to non-responses on the questionnaires. Sending reminders may have been a solution for this problem but would have interfered with the restricted time frame in which questionnaires had to be completed. Fifth, because of organizational reasons, the patients completed the baseline questionnaire at the end of the two PRISMA meetings rather than at the start. We realize this is inconsistent with RCTs, however, the two PRISMA meetings should be seen as a starting point to motivate/activate patients, with behavior change as the final objective. Because we were primarily interested in the outcomes during the months, the influence of the intervention on completing questionnaires before or after the intervention was less relevant. Sixth, a sample size calculation was not specifically performed for examining effects on the secondary outcomes for the present study [19].

## Conclusion

In previous observational research, the diabetes self-management education program PRISMA seemed to improve self-management behavior after 3 months. However, in this study, PRISMA did not improve self-reported outcomes (knowledge, skills and confidence for self-management, diabetes self-care behavior, health-related quality of life, and emotional well-being) after 6 or 12 months. In addition, it was not possible to make a statement about the clinical effects given the large number of missing values. The lack of effects on the outcomes in the current study does not automatically mean that PRISMA is not effective in improving self-management skills. Our target group may have been uninterested in this type of intervention, which is the reality of usual care. Therefore, additional research is necessary. In pragmatic trials such as the current study, it is essential to monitor all possible information during the implementation phase of the intervention. This will improve the reliability of the data collected.

## Data Availability

The datasets generated and/or analyzed during the current study are not publicly available because public access was not included in the informed consent, but are available from the corresponding author on reasonable request.

## Appendix

**Table 3:** Clinical outcomes (HbA1c, BMI, systolic blood pressure, cholesterol (total, HDL, LDL]) of the intervention and control group at 6 and 12 months

	Baseline – 0 months				RCT phase – 6 months					Extension of the RCT phase – 12 months				
Clinical parameters 푛 (%)/mean ± SD/median (25–75 quartiles)	Intervention group (*n* = 95)		Control group (*n* = 98)		Intervention group (*n* = 95)		Control group (*n* = 98)			Intervention group (*n* = 95)		Control group (*n* = 98)		
	Value	Missing	Value	Missing	Value	Missing	Value	Missing	Mean difference (95% CI)	Value	Missing	Value	Missing	Mean difference (95% CI)
HbA1c in mmol/mol	50.7 ± 8.5	70 (73.7)	54.7 ± 11.7	75 (76.5)	51.1 ± 11.0	71 (74.7)	58.2 ± 14.3	74 (75.5)	−7.08 (− 14.49–0.33)	53.91 ± 7.72	73 (76.8)	56.2 ± 10.3	82 (83.7)	− 2.28 (− 8.29–3.63)
BMI	28.5 ± 4.3	64 (67.4)	30.8 ± 5.1	66 (67.3)	28.5 ± 4.3	58 (61.1)	30.7 ± 5.3	44 (44.9)	−2.17 (− 4.25– − 0.09)*	29.9 ± 4.7	48 (50.5)	31.2 ± 6.3	50 (51.0)	− 1.29 (− 3.56–0.98)
Systolic blood pressure in mmHG	139.35 ± 15.0	58 (61.1)	135.59 ± 14.93	61 (62.2)	142.19 ± 17.56	59 (62.1)	139.54 ± 18.74	41 (41.8)	2.65 (− 5.09–10.39)	138.51 ± 13.35	50 (52.6)	137.94 ± 13.68	48 (49.0)	0.57 (−4.95–6.09)
Cholesterol in mmol/L	4.47 ± 1.45	78 (82.1)	4.08 ± 1.21	80 (81.6)	4.10 ± 0.97	77 (81.1)	3.92 ± 0.81	77 (78.6)	0.18 (− 0.40–0.76)	4.09 ± 0.90	80 (84.2)	4.26 ± 0.98	87 (88.8)	− 0.18 (− 0.94–0.59)
HDL in mmol/L	1.25 ± 0.3	78 (82.1)	1.06 ± 0.3	80 (81.6)	1.11 ± 0.31	77 (81.1)	1.26 ± 0.30	77 (78.6)	− 0.15 (− 0.34–0.05)	1.24 ± 0.39	80 (84.2)	1.10 ± 0.44	87 (88.8)	0.14 (− 0.2–0.48)
LDL in mmol/L	2.19 ± 1.7	78 (82.1)	2.27 ± 2.1	80 (81.6)	2.23 ± 0.92	77 (81.1)	2.01 ± 0.70	77 (78.6)	0.23 (− 0.31–0.77)	2.10 ± 0.56	80 (84.2)	2.17 ± 0.68	87 (88.8)	− 0.07 (− 0.58–0.43)

* Significant

## Abbreviations

BMI: Body mass index
DESMOND: Diabetes Education and Self-Management for Ongoing and Newly Diagnosed
EQ-5D-3 L: EuroQol Five Dimension
GP: General practitioner
LD: Low-density lipoprotein
PAM: Patient Activation Measure
PN: Practice purse
PRISMA: Proactive Interdisciplinary Self-Management
RCT: Randomized controlled trial
SDSCA: Summary of Diabetes Self-Care Activities
WHO-5: The World Health Organization Well-being Index 5-Item
T2DM: Type 2 diabetes mellitus
